# Editorial: Neonatal and Pediatric Cerebro-Cardio-Pulmonary Resuscitation (CCPR): Where Do We Stand and Where Are We Heading?

**DOI:** 10.3389/fped.2018.00165

**Published:** 2018-06-04

**Authors:** Utpal Bhalala, Graeme Polglase, Eugene Dempsey

**Affiliations:** ^1^Department of Pediatrics, The Children's Hospital of San Antonio, Baylor College of Medicine, Houston, TX, United States; ^2^Department of Obstetrics and Gynaecology, Monash University, Melbourne, VIC, Australia; ^3^Department of Paediatrics, University College Cork, Cork, Ireland

**Keywords:** neonatology, cardio-pulmonary resuscitation, therapeutic hypothermia, neonatal asphyxia, cerebral cortex

An overwhelming response followed my inaugural article in Frontiers in Pediatric Critical Care in 2015, which focused on hypoxic ischemic injury in the developing brain (1). My article sparkled a renewed interest in neonatal and pediatric cerebro-cardio-pulmonary resuscitation (CCPR). In reviewing the research investment into neonatal deaths, only 10% of research expenditure is directed toward 90% of the world's global burden of disease—the so called “10/90 gap” (2). The Millennium Development Goal to reduce the under-five mortality by two-thirds by 2015 was not achieved (3), and neonatal death remains a significant contributor to this category of childhood mortality (4).

To address these concerns, we gathered a team of neonatal and pediatric CCPR experts from across the globe and proposed a research topic on neonatal and pediatric CCPR. The topic received a robust response from across the world and we received original research articles and review articles from neonatal and pediatric resuscitation experts.

Whilst prolonged mechanical ventilation has been well established to induce injury to preterm brain, ventilation-induced brain injury may occur as early as the initiation of ventilation in the delivery room and seems to be a potentially preventable contributor to brain injury (5). An original investigation conducted by Alahmari et al in our research topic tested the hypothesis that neuropathology associated with *in utero* inflammation is exacerbated by inappropriate delivery room ventilation in preterm lambs (12). The findings of the study not only supported the concept of neuroinjury in preterm babies worsened by hyperventilation in the delivery room but it also defined neuroimaging changes and neuroprotective ventilation strategies in the delivery room. Using a neonatal resuscitation simulator, Solveg and co-authors investigated volume-controlled delivery room ventilation (5). The study compared a new volume-controlled resuscitator (The Next Step^TM^) with four other available devices used for stabilization in the delivery room. The study showed that routinely used newborn resuscitators delivered excess tidal volume, whereas the Next Step^TM^ under delivered in the low compliant test lung (5). These findings published clearly bridge important knowledge gaps in the area of delivery-room ventilation and its impact on neurologic outcome in preterm infants.

While studying an optimum technique and technology to deliver safe ventilation in the delivery room, Solveg and co-authors also raise an equally important question on optimal chest compression (CC) rate and compression to ventilation (C:V) ratio during delivery room resuscitation (5, 6). Despite a lack of scientific evidence supporting CC at 90/min and C:V ratio of 3:1, the investigation of alternative CC interventions in human neonates is ethically challenging. Also, the infrequency of CPR in the delivery room make randomized controlled trials difficult to perform. Therefore the physiology of CPR has been investigated in animal and mannequin models. Neither continuous CCs with synchronous ventilation nor higher C:V ratios of 9:3 or 15:2 have been associated with better outcomes as compared to C:V ratio of 3:1 in neonatal resuscitation (6, 7). Infact, uninterrupted or less interrupted CCs have been associated with poor quality CCs due to provider fatigue (8, 9) The authors appropriately bring up un-answered questions as potential future directions—biomechanical aspect of CPR using mechanical/automated devices and feedback systems during neonatal CPR have not been explored. Also, video recording of delivery room CPR and outcomes have yet to be reported (6).

On one hand, the investigators are looking at post-partum measures such as placental transfusion to improve outcomes of neonatal asphyxia (7) and on the other hand, investigators are exploring antepartum measures such as maternal dietary supplements to support fetal metabolism and reduce severity of organ injury caused by intrapartum asphyxia (8). In their review article, Katheria and co-authors discuss the current evidence on placental transfusion in neonates requiring resuscitation. Placental transfusion using techniques such as delayed cord clamping or cord stripping or milking have been described over last several decades (10, 11). Though RCTs on these techniques have shown beneficial effects, a number of logistic challenges such as maintaining sterile field and ergonomic difficulties of providing effective ventilation while attached to the placenta need exploration. Future trials on effects of placental transfusion on outcome will help determine guidelines on use of placental transfusion during delivery room resuscitation, especially in preterm newborns. LaRosa and colleagues provide an extensive review of existing information on organ injury following intrapartum asphyxia. The authors also shed light on maternal therapies, specifically creatine, to minimize organ injury related to intrapartum asphyxia (8). Prior animal studies have shown promising results on neonatal mortality and protection of organs such as brain, diaphragm, kidney and skeletal muscles after maternal dietary supplementation with creatine during antepartum period (12, 13). There is a need for bench-to-bedside research exploring the role of creatine in pregnant women (14).

Current guidelines recommend monitoring of vital signs which do not include monitoring of the brain, the most vulnerable organ especially to hypoxia during transition immediately after birth. Doppler sonography allows non-invasive monitoring of the perfusion in different regions of the brain. However, this method is limited since it does not allow continuous monitoring, it is difficult during transition after birth and it is prone to motion artifacts, especially in infants (15). In the last decade, several studies have demonstrated feasibility of cerebral oxygenation monitoring using Near Infrared Spectroscopy (NIRS) during immediate transition in healthy term neonates (16–21), preterm neonates (22, 23), and even during resuscitation with chest compressions (24). Cerebral oxygenation changes within the first minutes after birth do not correlate with arterial oxygen saturation, heart rate, or even peripheral tissue oxygenation (20, 25). The authors discuss the studies on cerebral tissue oxygenation in relation to interventions such as delayed cord clamping and sustained lung inflation. Monitoring cerebral tissue oxygenation with NIRS and interventions to correct the cerebral oxygenation changes as detected by NIRS can reduce cerebral hypoxia. A large randomized controlled trial to investigate the effect of cerebral NIRS monitoring in combination with intervention guidelines on short- and long-term outcomes in preterm neonates is currently underway.

Dix et al in their review paper discuss about the importance of cerebral oxygenation monitoring in the first 3 days of life, particularly during neonatal resuscitation and beyond resuscitation (10). Apart from discussing the evidence on cerebral oxygenation monitoring during interventions such as ventilation, management of hypotension and during neonatal surgery, they also discuss the findings of a large international randomized controlled trial, the SafeboosC study (Safeguarding the brains of our smallest children). The percentage of time spent outside a predetermined “normative” range of rScO2 (55–85%), was significantly lower in the group with NIRS monitoring as compared to the blinded control group (median 36.1 vs. 81.3%) (26). The authors also highlight the current evidence on low cerebral oxygenation and/or abnormality of cerebral autoregulation as measured by NIRS and poor neurologic outcomes in neonates. More data is needed to define how assessment of neonatal brain oxygenation could guide clinical management, prevent brain injury and provide important information regarding the infant's prognosis.

Vesoulis and co-authors present a very comprehensive review on techniques of assessing cerebral autoregulation and current evidence on role of cerebral autoregulation in the pathogenesis of brain injury in premature infants (11).

The next steps are assessing a cerebral autoregulation quantification system, which is a hybrid of currently available techniques, and neuroprotective interventions directed toward autoregulation targets. Due to ischemia-reperfusion, neuronal injury continues beyond resuscitation. It is therefore important to understand cerebral perfusion and autoregulation in post-resuscitation period. Monitoring of cerebral perfusion and management of disturbance of cerebral autoregulation are important not only during resuscitation but also in the post-resuscitation period. Lordanova and colleagues discuss changes in cerebral blood flow during post-arrest period and methods to assess cerebral perfusion during post-resuscitation period (15). There has been a growing interest in use of NIRS for non-invasive monitoring of the cerebral perfusion. Though transcranial doppler is a promising modality to monitor cerebral blood flow after resuscitation, it is not well utilized in clinical practice. Though monitoring of cerebral blood flow and oxygenation are crucial during and beyond resuscitation, a multimodality monitoring incorporating simultaneous monitoring of blood flow, oxygenation and electroencephalogram is more likely to provide a global status of neurologic function during and after resuscitation.

The EEG is exquisitely sensitive to any impairment in oxygen delivery to the brain. A reduction in oxygen leads to an immediate suppression of synaptic transmission with a reduction (often complete suppression) in EEG amplitude. A sustained change in the EEG signals a risk of impending brain injury. EEG activity should recover immediately following restoration of oxygen delivery to the brain, if EEG activity does not return immediately post-resuscitation or activity is severely disrupted, this indicates that the infant is at risk of hypoxic-ischemic brain injury. Dr. Finn and co-authors describe the rationale for using delivery room EEG, explored the current evidence on feasibility of delivery room EEG monitoring and the role of DR EEG monitoring on neurologic outcome. Though delivery room EEG is feasible, it is challenging to apply within minutes after delivery. The EEG pattern correlates with cerebral oxygen saturation and oxygen extraction and therefore it provides useful information about neonates' status during transition after delivery. This could potentially identify neonates who might benefit from early cooling within first 3 h of delivery. Prospective, randomized controlled studies are needed to support the authors' statements on use of EEG in delivery room, especially to determine early (<3 h after delivery) vs. late (3–6 h after delivery) cooling.

In summary, the field of cerebro-cardiopulmonary resuscitation is evolving significantly but many questions still remain unanswered. There is a growing interest in monitoring of cerebral function through non-invasive modalities such as NIRS and understanding the effects of interventions such as placental transfusion, ventilation and use of epinephrine during neonatal resuscitation. Multi-center, prospective, randomized controlled trials focused on multi-modality monitoring of brain function and goal-directed interventions will potentially help us bridge the knowledge gaps.

## Author contributions

All authors listed have made a substantial, direct and intellectual contribution to the work, and approved it for publication.

### Conflict of interest statement

The authors declare that the research was conducted in the absence of any commercial or financial relationships that could be construed as a potential conflict of interest.
